# An Oil Hyper-Accumulator Mutant Highlights Peroxisomal ATP Import as a Regulatory Step for Fatty Acid Metabolism in *Aurantiochytrium limacinum*

**DOI:** 10.3390/cells10102680

**Published:** 2021-10-06

**Authors:** Etienne Deragon, Martin Schuler, Riccardo Aiese Cigliano, Younès Dellero, Gregory Si Larbi, Denis Falconet, Juliette Jouhet, Eric Maréchal, Morgane Michaud, Alberto Amato, Fabrice Rébeillé

**Affiliations:** 1 Laboratoire de Physiologie Cellulaire et Végétale, Université Grenoble Alpes, CNRS, CEA, INRAE, CEDEX 9, 38054 Grenoble, France; etienne.deragon@cea.fr (E.D.); martin.schuler@outlook.com (M.S.); younes.dellero@inra.fr (Y.D.); gregory.si-larbi@cea.fr (G.S.L.); denis.falconet@cea.fr (D.F.); juliette.jouhet@cea.fr (J.J.); eric.marechal@cea.fr (E.M.); morgane.michaud@cea.fr (M.M.); 2 Sequentia Biotech SL, Carrer Pamplona, 08018 Barcelona, Spain; raiesecigliano@sequentiabiotech.com; 3 Institute of Genetic, Environment and Plant Protection, UMR 1349 IGEPP INRA, Agrocampus Ouest Rennes, Université Rennes 1, Domaine de la Motte BP35327, CEDEX, 35653 Le Rheu, France

**Keywords:** triacylglycerol (TAG), thraustochytrids, ω3-docosahexaenoic acid (DHA), lipid metabolism, β-oxidation, peroxisomal adenylate transporter

## Abstract

Thraustochytrids are marine protists that naturally accumulate triacylglycerol with long chains of polyunsaturated fatty acids, such as ω3-docosahexaenoic acid (DHA). They represent a sustainable response to the increasing demand for these “essential” fatty acids (FAs). Following an attempt to transform a strain of *Aurantiochytrium limacinum*, we serendipitously isolated a clone that did not incorporate any recombinant DNA but contained two to three times more DHA than the original strain. Metabolic analyses indicated a deficit in FA catabolism. However, whole transcriptome analysis did not show down-regulation of genes involved in FA catabolism. Genome sequencing revealed extensive DNA deletion in one allele encoding a putative peroxisomal adenylate transporter. Phylogenetic analyses and yeast complementation experiments confirmed the gene as a peroxisomal adenylate nucleotide transporter (*AlANT1*), homologous to yeast *ScANT1* and plant peroxisomal adenylate nucleotide carrier *AtPNC* genes. In yeast and plants, a deletion of the peroxisomal adenylate transporter inhibits FA breakdown and induces FA accumulation, a phenotype similar to that described here. In response to this metabolic event, several compensatory mechanisms were observed. In particular, genes involved in FA biosynthesis were upregulated, also contributing to the high FA accumulation. These results support AlANT1 as a promising target for enhancing DHA production in Thraustochytrids.

## 1. Introduction

*Aurantiochytrium limacinum* is an obligate heterotrophic marine unicellular protist belonging to the Thraustochytrid family. Thraustochytrids are present almost everywhere in the oceans, from tropical to polar areas, and from the surface down to 2000 m below sea level [1,2,3,4]. Because they are obligate heterotrophs, Thraustochytrids are more abundant in habitats containing decaying biological material, such as superficial sediment layers, mangroves, or river effluents [3]. In the past decade, Thraustochytrids have attracted biotechnological interest because they naturally accumulate high levels of triacylglycerols (TAGs). TAGs represent a valuable source of fatty acids (FAs) for either human and animal health or green chemistry purposes [5,6]. The peculiarity of Thraustochytrids compared with many other microalgae is their strikingly high content of very long chain polyunsaturated fatty acids (VLCPUFA), mainly ω3-docosahexaenoic acid (DHA, 22:6) [7,8,9,10,11]. DHA is synthesized at extremely low levels in animals and is therefore considered as a ‘conditionally essential’ FA [12], which means it must be obtained from the diet. In humans, DHA accumulates in the brain and is required for the good visual and neural development in infants [13]. Presently, the most widely and naturally available dietary source of ω3-VLCPUFAs is fish oil. However, overexploitation of fish stocks and their contamination by toxic substances such as heavy metals impose us to find alternative and more sustainable sources [14]. For the reasons depicted above, Thraustochytrids are emerging models for fundamental and applied research.

FA synthesis in Thraustochytrids is more complex than in many other marine protists because it involves two different pathways that operate independently [6]. The first pathway requires a type I Fatty Acid Synthase (FAS) system similar to that found in animals and produces FA chain length of 16 carbons (16C). The second pathway involves a Polyketide Synthase-like (PKS-like) machinery, or PUFA synthase, to produce VLCPUFAs of 20C and 22C [15,16,17,18]. Many efforts and many different strategies are currently being undertaken to elucidate how the two pathways are regulated and coordinated, and how the lipid production could be improved in Thraustochytrids. Indeed, as already reviewed [5,6], the literature is abundant about the impact of culture conditions, or about attempts of metabolic engineering based on genetic modifications. To date, most of the molecular approaches attempted to improve the VLCPUFA production by targeting anabolic pathways such as those involved in FA synthesis, including the FAS system [19,20] and the PUFA synthase complex [21,22,23], or those involved in glycerolipid synthesis [24,25]. FA catabolism is also a potential target to enhance the lipid production. Genomic and qPCR analyses of *Aurantiochytrium limacinum* and *Hondaea fermentalgiana* [11,26] indicate that FA oxidation in Thraustochytrids occurs in both mitochondria and peroxisomes. FA catabolism first requires detaching the FAs from glycerolipids (TAGs), transporting them into peroxisomes, and activating them in the form of acyl-CoAs to allow their oxidation by the β-oxidation cycle. The products of the peroxisomal β-oxidation are then shuttled to mitochondria for a complete oxidation into CO_2_ and H_2_O [26,27,28]. To our knowledge, there is, so far, only one publication exploring the catabolic side of FA metabolism in *Aurantiochytrium*. The authors showed that disrupting the genes coding for acyl-CoA oxidases resulted in a higher FA productivity [29].

Genetic transformation by biolistic (i.e., using particle bombardment) offers the advantage to deliver any form of RNA, DNA, or protein in almost any type of cell, including those with thick walls or silica walls such as diatoms [30]. This relatively simple-to-use method is widely used to transform plants [31], which prompted specific efforts to estimate the genetic instability and unintended consequences resulting from such approaches. Indeed, biolistic violently integrates DNA, in a rather random way. Although some transgenic events appear as simple insertions with no other evident genome damage, others show serious genome damages including chromosome breakages and large deletions [32] or transpositional activation [33]. Thus, a successful biolistic transformation also relies on a combination of various and complex mechanisms of DNA repair. A corollary is that the process for biolistic transformation can be also used for the production of some random mutations.

In the present work, following a biolistic attempt to transform a strain of *Aurantiochytrium limacinum* (CCAP 4062/1) [11], we isolated by serendipity a clone that did not integrate any recombinant DNA but displayed two to three times more FAs than the original strain. Using metabolic, transcriptomic, and genomic analyses, we identified the biolistic side effects at the origin of this phenotype. The results pointed out a potential target indirectly involved in FA oxidation, which could significantly increase the lipid content in *A. limacinum* when down regulated.

## 2. Materials and Methods

### 2.1. Aurantiochytrium limacinum Strain, Culture Media, and Growth Experiments

*Aurantiochytrium limacinum* CCAP 4062/1 strain was collected in Mayotte (Indian Ocean, 12°48′51.8″ S, 45°14′21.7″ E) and routinely cultivated at 20 °C in 250-mL Pyrex^®^ Erlen–Meyer flasks filled with 50 mL of rich (R) or poor (P) media [11] with a 100-rpm orbital shaking. P medium is an R medium containing 40 times less glucose and yeast extract. For all experiments, 6-day-old axenic cultures grown on R were transferred to fresh culture media at an initial cell concentration of 5 × 10^5^ cells·mL^−^^1^. Growth was monitored following the increase of DW. To estimate the role of β-oxidation in *A. limacinum* growth, the glucose carbon source in the R medium was replaced by 2 mM palmitic acid (C16:0). Stock solutions of palmitic acid were made in 100% TWEEN 80 at a concentration of 50 mM). Two-day-old cells in the exponential phase of growth were centrifuged at low speed and then transferred to glucose-free medium for 24 h to remove all glucose resulting from the subculture process. Cells were then transferred to new glucose-free medium containing 2 mM C16:0 at a final concentration of 5 × 10^5^ cells·mL^−^^1^. The growth was followed by measuring the DW increase over 3 days.

### 2.2. Lipid Analyses

Lipids were extracted according to Folch et al. [34]. FAs were converted into methyl esters (FAME), then analyzed by GC–MS/FID (Clarus 80, Perkin Elmer, Waltham, MA, USA) on a BPX70 (SGE) column, as previously described [11,35]. FAMEs were identified by comparison of their retention times with those of standards (Sigma, Saint-Louis, MO, USA) and by their mass fragmentation spectra. They were quantified using C21:0 as internal standard.

### 2.3. Genomic, Transcriptomic, and qRT-PCR Analyses

Nucleic acid extraction, sequencing, and bioinformatics methods are described in Morabito et al. [36] and in Appendix A. RNA samples from 3-day-old cells were reverse transcribed using the SuperScript IV VILO Mastermix with ezDNAse kit (ThermoFisher, Waltham, MA, USA) according to the manufacturer’s instructions. The qPCR reactions were run in a final volume of 10 µL containing 20 ng of cDNA, 5 µL of Power SYBR^®^ Green PCR Master Mix (Applied Biosystems, ThermoFisher), and 600 nM of each primer. Reactions were performed in a CFX ConnectTM Real-Time System (BioRad, Hercules, CA, USA) with the following program: initial denaturation step at 95 °C for 10 min and 40 denaturation-amplification-elongation cycles (95 °C, 10 s; 60 °C, 10 s; 72 °C, 30 s), followed by melting curve assessment (65 °C to 95 °C, with a 0.5 °C increment). All primer sequences are available in the Supplementary Data and Methods file. Two sets of primers were used to estimate the expression level of *AlANT1* (e_gw1.9.603.1) at day 3, targeting two different regions of the gene. Transcript levels were normalized against the Cystein desulfurase *NFS1* (estExt_fgenesh1_kg.C_30063) gene. *NSF1* expression did not vary in the WT or in LAS strains in RNA-seq experiment. The differential expression analyses and statistics were performed using the Pair Wise Fixed Reallocation Randomisation Test method developed in the Relative Expression Software Tool REST^©^ [37]. Biological triplicates and technical triplicates of each reaction were performed.

### 2.4. Yeast Cultures and Transformations

The isogenic *Saccharomyces cerevisiae* strains WT (BY4741: MATa ura3 leu2 his3 met15) and *Δant1* (YPR128C: MATa ura3 leu2 his3 met15 ANT1::KANMX) used in this study were kindly provided by Dr. William Prinz (NIH, Bethesda, MD, USA). After transformation, all the strains described in the present work were grown on synthetic media containing 6.7 g·L^−^^1^ of Yeast Nitrogen Base (YNB, MP Biomedicals, Illkirch-Graffen-Staden, France), 0.77 g·L^−^^1^ of CSM-URA (MP Biomedicals). The carbon sources were either 20 g·L^−^^1^ glucose or 2 mM lauric acid (C12:0). Lauric acid was dissolved in 100% TWEEN 80 at a concentration of 50 mM for stock solution. Growth was monitored following the increase of OD at 600 nm.

The mutant *Δant1* was complemented with either *ScANT1*, g11073, or the truncated form of g11073. For the transformations, the DNA fragments were in vivo cloned by homology into a PstI-BamHI-digested YEplac195 backbone under the control of the promoter PMA1 and with the ADH1 terminator. A 6× His tag was cloned in frame with the genes of interest in 3′, upstream and in frame with the stop codon. First, the promoter PMA1, terminator ADH1, and coding sequences of *ScANT1*, g11073, and truncated g11073 were amplified by PCR. Primers are listed in the Supplementary data and methods file. Yeast cells were directly transformed using the lithium acetate method [38] in the presence of the digested plasmid and the purified PCR fragments, as fully described in the Supplementary data and methods file. Transformed cells were selected in CSM plates without URA. Positive clones were grown in CSM medium. Plasmids were extracted from yeast cells with the kit ZymoPrep D2004, (Zymo Research, Irvine, CA, USA), amplified in DH5α competent cells, and then sequenced for validation. Complemented yeast strains and WT were inoculated in biological triplicate at an initial OD of ca. 0.15 in 50 mL of fresh medium in 250-mL Pyrex^®^ Erlen–Meyer flasks. Cultures were incubated at 30 °C under agitation at 250 rpm.

### 2.5. Sample Preparation for Electronic Microscopy

Samples were prepared as previously described [39]. Briefly, cells were fixed in 0.1 M phosphate buffer (PB) (pH 7.4) containing 2.5% (*v/v*) glutaraldehyde for 2 h at room temperature and then stored overnight at 4 °C. Samples were then washed five times in 0.1 M PB (pH 7.4). Samples were fixed by a 1-h incubation on ice in 0.1 M PB (pH 7.4) containing 2% osmium and 1.5% ferricyanide potassium before they were washed five times with 0.1 M PB (pH 7.4). Samples were resuspended in 0.1 M PB (pH 7.4) containing 0.1% tannic acid and incubated for 30 min in the dark at room temperature. Samples were again washed five times with 0.1 M PB (pH 7.4), dehydrated in ascending sequences of ethanol, and infiltrated with ethanol/Epon resin mixture. Finally, the samples were embedded in Epon. Ultrathin sections (50–70 nm) were prepared with a diamond knife on a PowerTome ultramicrotome (RMC products, Tucson, AZ, USA) and collected on 200-µm nickel grids. Samples were visualized by scanning transmission electron microscopy (STEM) using a MERLIN microscope (Zeiss, Oberkochen, Germany) set up at 30 KV and 240 pA.

### 2.6. Phylogenetic Analyses

The nucleotide coding sequence of the g11073 locus was in silico translated and the obtained amino acid sequence was aligned in the data set by Arai et al. [40] The alignment was performed using a customized pipeline in NGphylogeny.fr [41] using MUSCLE v3.8.1551 software. The ambiguously aligned regions were curated using the Block Mapping and Gathering with Entropy (BMGE v1.12_1) software implemented in NGphylogeny.fr using default settings. MEGA X v10.0.5 software was fed with the aligned and curated data set. The best evolutionary model was evaluated and a Maximum Likelihood phylogenetic analysis was performed. In order to define the best evolutionary model, MEGA X was used to compare the 56 models implemented. The LG+G model was chosen (lowest Bayesian Information Criterion (BIC) score). The phylogeny was inferred by Maximum Likelihood with 2000 bootstrap pseudoreplicates. The tree with the highest log likelihood (−10,454.51) was retained. Initial trees for the heuristic search were obtained by applying the BioNJ method to a matrix of pairwise distances estimated using a JTT model. A discrete Gamma distribution was used to model evolutionary rate differences among sites (five categories (+G, parameter = 2.9679)).

## 3. Results

We attempted to transform *Aurantiochytrium limacinum* CCAP 4062/1 [11] to produce a zeocin-resistant strain. A commercial pUC19 vector was modified to introduce the *ShBle* gene under the control of the promoter and terminator sequences of an endogenous polyubiquitin gene. Following a biolistic transformation, potentially transformed clones were selected on agarose plates containing 200 µg mL^−1^ zeocin. One of the selected clones appeared later on as a false positive since the initial zeocin-resistant phenotype disappeared after a few subculturing steps. In addition, PCR screening revealed no fragments corresponding to the *ShBle* gene or any part of the vector plasmid. Whole genome sequencing confirmed the absence of any exogenous DNA. However, this clone showed a strong lipid accumulation phenotype, with a higher lipid concentration than the WT strain. This clone was termed LAS, for Lipid Accumulating Strain.

### 3.1. Growth and Fatty Acid Content of LAS and WT

**Figure 1:**
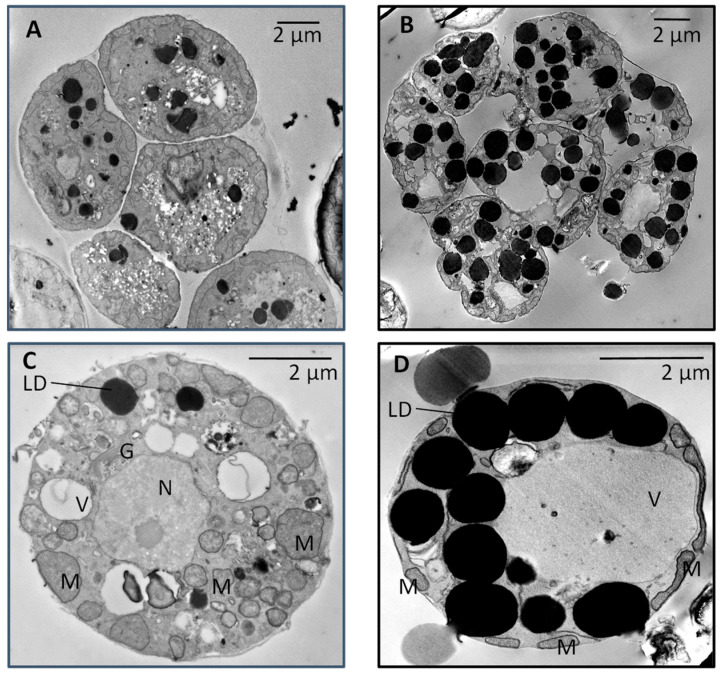
Scanning transmission electron microscopy (SEM) pictures of *Arantiochytrium limacinum* (**A**,**C**) 2-day-old culture of the WT; (**B**,**D**) 2-day-old culture of the LAS mutant. (**A**,**B**) show a group of cells, and (**C**,**D**) show the detailed structures of a single cell. The oil bodies appear as black spots. LD, lipid droplets; V, vacuole; M, mitochondria; N, nucleus; G, Golgi apparatus.

**Figure 2:**
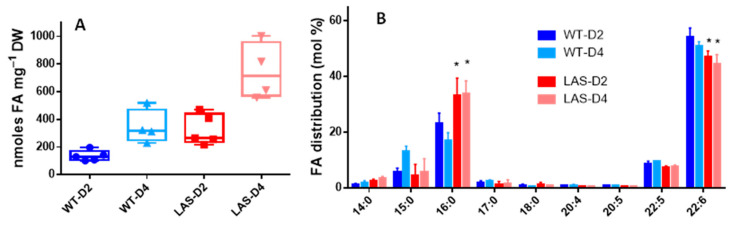
Analyses of FAs. (**A**) Box plots showing total FA content in WT (in blue) and LAS (in red) on day 2 (D2, end of the exponential growth phase) and day 4 (D4, stationary phase). The experiments were carried out over a period of 1 year. These Tukey representations show all points from min to max. Each point represents a different experiment and is the average of three biological replicates. (**B**) FA profiles in WT (blue bars) and LAS (red bars) on day 2 (D2) and day 4 (D4). Error bars indicate standard deviation for five (D2) and four (D4) independent experiments. Stars indicate statistical differences versus WT (*t* test, *p* value < 0.05). Graphs and statistics were done with GraphPad Prism^®^ v7.00 software.

The WT and LAS growth curves were similar during the first 2 days corresponding to the exponential phase of growth [11] (Supplementary Figure S1). Thereafter, the biomass (expressed as DW·mL^−1^) increased more in LAS than in WT, likely because the former accumulated significantly more lipids (TAGs) than the latter. Indeed, as shown in Figure 1A–D, scanning transmission electron microscopy (STEM) clearly showed that LAS contained more lipid droplets than WT. This was confirmed by FA analyses (Figure 2A), with LAS displaying 2–3 times more FAs than WT, either at the end of the exponential phase of growth (Day 2) or later in the stationary phase (Day 4). In addition, the FA profile in LAS revealed significant differences compared with WT (Figure 2B); the proportion of C16:0 in LAS was higher than in WT, whereas the proportion of C22:6 was lower. Then, the ratio of FA chains synthesized by the FAS system (C14:0 + C15:0 + C16:0) to FA chains synthesized by the PUFA synthase (C22:5 + C22:6) was monitored throughout growth.

**Figure 3:**
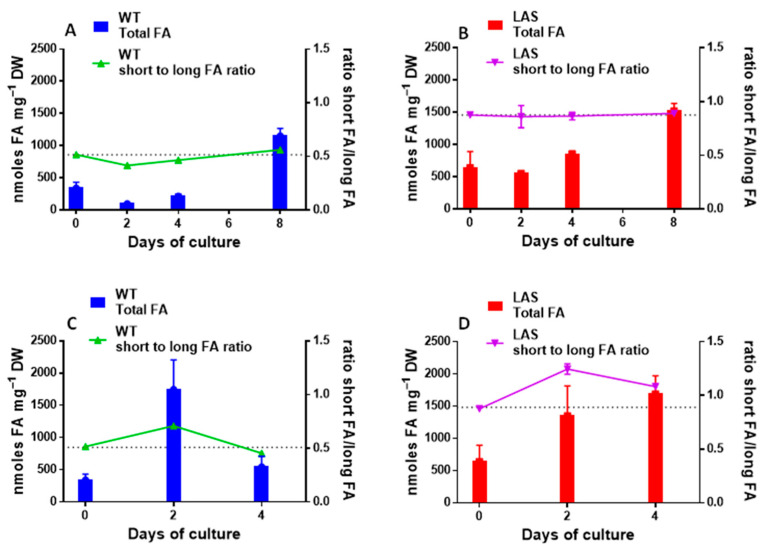
Effect of the medium composition on FA accumulation and short to long FA ratio in WT (blue bars) and LAS (red bars). (**A**,**B**) rich (R) medium; (**C**,**D**) poor (P) medium. The short to long FA ratio (right Y axis) represents (14:0 + 15:0 + 16:0)/(22:5 + 22:6). The broken line shows the initial ratio recorded at the beginning of the experiment. Each value is the average of three biological replicates and error bars indicate standard deviation. Graphs and statistics were done with GraphPad Prism^®^ v7.00 software.

It was previously observed [35] that cells inoculated in a rich (R) medium first consumed their TAGs to sustain their rapid growth, and then, upon nitrogen limitation, started to accumulate TAGs at the expense of glucose to build up new storages. As shown in Figure 3A, the total amount of FAs in WT decreased 3–4 times during the first 2 days. This was associated with a decrease of the short to long FA ratio, suggesting that shorter FAs, essentially C16:0, were more rapidly consumed than longer ones (C22:6). This ratio returned to the initial value with the accumulation of newly synthesized FAs. In LAS (Figure 3B), the total amount of FAs decreased less after 2 days of culture, suggesting a slower consumption of storage lipids. Concomitantly, the short to long FA ratio remained high throughout the experiment. In a poor (P) medium, nitrogen was limiting right at the beginning of the experiment and TAGs were immediately synthesized in WT until glucose was exhausted [35] (Figure 3C). Then, after 2 days, TAGs were consumed to sustain the energy demand. Here again, the short to long FA ratio in WT was modified during the initial FA accumulation phase, suggesting that C16:0 was more rapidly synthesized than C22:6. It declined back to the initial value with the consumption of FAs. In LAS (Figure 3D), the FA level also increased after 2 days but remained high even after 4 days. This suggests that FAs were poorly utilized to sustain cell basal activity, unlike WT. The short to long FA ratio increased after 2 days and stayed high thereafter. Taken as a whole, these results strongly suggest a slower catabolism of FAs in LAS compared with WT, a metabolic change that also affects the short to long FA ratio.

**Figure 4:**
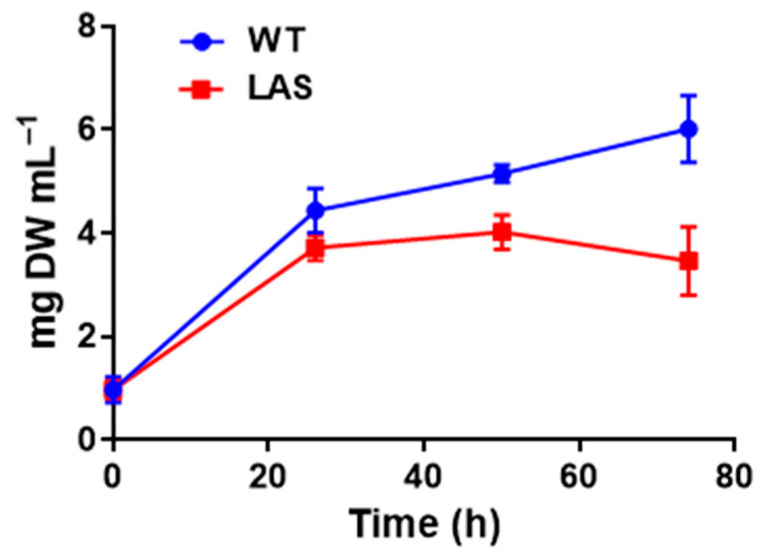
Growth of WT (in blue) and LAS (in red) in medium containing C16:0 as carbon source. Each value is the mean of three biological replicates, and error bars indicate the standard deviation. Graphs and statistics were performed with GraphPad Prism^®^ software.

To validate this hypothesis, C16:0 replaced glucose as carbon source in the growth medium (Figure 4). LAS was not able to grow after 24 h, whereas WT did, although at a slower pace than in the presence of glucose (Supplementary Figure S1). The growth recorded during the first 24 h was probably due to minor sources of carbon present in the medium, such as the amino acids contained in the yeast extract.

### 3.2. Transcriptomic and Genomic Analyses of LAS

To get clues about the modifications affecting the lipid metabolism in LAS, transcriptomic (Illumina RNA-seq) and genomic (PacBio sequencing) analyses on LAS and WT were carried out. The transcriptomic and genomic sequencing data are accessible from the following references: Morabito et al., 2020 [36] and Appendix A. The complete list of differentially expressed (DE) genes involved in lipid metabolism is shown in the Supplementary Table S1. In Table 1 the DE genes involved in FA and glycerolipid metabolisms (LAS versus WT, Log_2_ fold change > |1|) are shown.

Few genes were significantly DE, most of them were upregulated. Among them, upregulation of genes involved in FA biosynthesis (PUFA synthase and FAS) could possibly explain the FA increase observed in LAS. However, none of them could explain the lower FA catabolism suggested by our experiments. Indeed, none of the genes involved in glycerolipid degradation (phospholipases, DAG and TAG lipases), transport of FAs into the peroxisomes (peroxisomal ABC carriers), esterification of FAs (acyl-CoA synthetases/ligases, including three putative acyl-CoA synthetases displaying the peroxisomal targeting signal—SKL), and β-oxidation were significantly downregulated (Table 1 and Supplementary Table S1). On the contrary, some of them were upregulated, suggesting a complex metabolic response.

To elucidate what happened during the biolistic transformation experiment, we searched for potential structural genomic variations, i.e., for variants displaying insertions/deletions (InDels) > 40 bp. The LAS and WT PacBio reads were compared to identify background variants not due to true biological variation or to the noise of the sequencing technology. About 6400 variants were found in the two samples, but only 13 were specific to LAS, proving the effectiveness of this approach (Appendix A). The filtered variants were manually inspected to exclude additional false positives, eventually producing a final list of five variants (Supplementary Table S2). Three corresponded to unexpressed genes in WT as well as LAS. Of the remaining genes, the locus g1125 encoded for a protein containing a putative transmembrane PAN_1 domain and carried a 46-bp insertion in the promoter region. Proteins containing a PAN_1 domain are found in many organisms, including parasites such as *Toxoplasma gondii* or viruses. The PAN_1 domain has functional versatility fulfilling diverse biological roles by mediating protein–protein and protein–carbohydrate interactions. In human, PAN_1 domains are present in hepatocyte growth factors, in plasminogen and coagulation factors [42,43]. In *Saccharomyces cerevisiae*, PAN_1 is present in an actin-cytoskeleton-associated protein likely involved in protein–protein interactions essential for endocytosis [44]. To our knowledge, no protein containing a PAN_1 domain is involved in lipid metabolism. Surprisingly, a BLAST search against the Aurli1 assembly (the reference genome for *Aurantiochytrium limacinum*) and the NCBI database did not produce any match with g1125.

**Figure 5:**
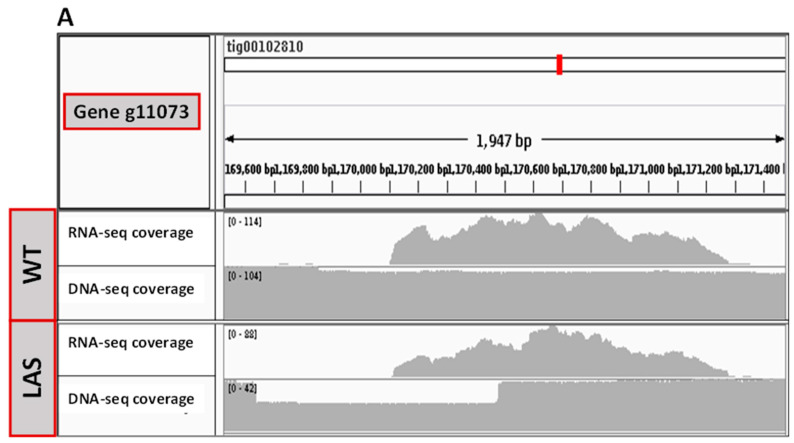

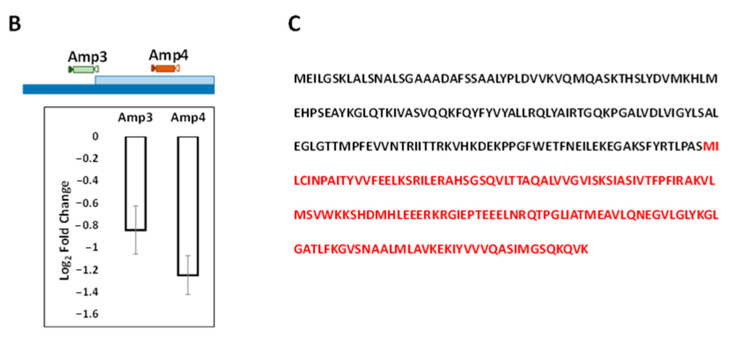
Genomic and transcriptomic analyses of g11073. (**A**) Genomic and transcriptomic analyses of the region containing the g11073 gene with Canu assembler software (Canu v1.9). This analysis shows the coverage of aligned short reads (Illumina) for RNA-seq and the coverage of aligned long reads (PacBio) for DNA-seq. (**A**) A 2-fold decrease of the DNA-seq coverage was recorded in the 5′ region of the locus in LAS, but not in WT, indicating important biolistic damages. (**B**) The qRT-PCR analysis of g11073 expression level in LAS versus WT at day 3, using two different amplicons (Amp3 and Amp4). The level of expression in WT is set at 0. (**C**) In silico translation of the g11073 gene. The putative truncated protein is in red, starting from the first methionine following the deletion.

The locus g11073 encoded for a protein predicted to belong to the ‘mitochondrial carrier’ family, possibly a ‘peroxisomal adenine nucleotide carrier’ (jgi|Aurli1|76398|e_gw1.9.603.1). In the reference genome Aurli1, the coding sequence annotation was shorter than g11073, possibly missing the first methionine (Supplementary data and methods file). As shown in Figure 5A, the coverage of aligned PacBio reads displayed a gap in the coding region of g11073 in LAS, but not in WT. The gap corresponded to a 50% decrease in coverage, suggesting that only one allele was affected. Thus, the g11073 sequence in LAS appeared truncated, showing a heterozygous 843-bp deletion in the promoter/gene body region of one of the two alleles. About 400 bp were missing upstream of the coding sequence and 440 bp were missing within the 5′-end of the gene. A 2-fold downregulation of g11073 (Amp3 Log_2_FC = −0.84 ± 0.22, Amp4 Log_2_FC = −1.25 ± 0.18) in LAS vs. WT was recorded by qRT-PCR, suggesting that the truncated allele was not or only minimally expressed (Figure 5B). If expressed, however, it can be estimated from these data that the size of the truncated protein would approximately be half of the native form (Figure 5C).

The contribution of the peroxisomal ATP carrier to FA catabolism is reported for several organisms, including plants and yeast. In *Saccharomyces cerevisiae*, disruption of the peroxisomal adenylate carrier ANT1 (YPR128c) resulted in an impaired growth when medium chain FA laurate (C12:0) was provided as the only carbon source [45,46], and the same holds true for *Candida boidinii* [47]. In the oleaginous yeast *Yarrowia lipolytica*, a *ΔYlant1* mutant contained 20% more FAs on a dry weight basis than the wild type [48]. In plants, Arabidopsis lines repressing the two *PNC* genes displayed an impaired peroxisomal ATP import associated with a 10-fold increase of all FAs [49]. Because the peroxisomal adenylate carrier plays an important role in plant and yeast β-oxidation, we hypothesized that the deletion event recorded in LAS might be responsible for the observed lipid phenotype.

### 3.3. Functional Characterization of g11073

**Figure 6:**
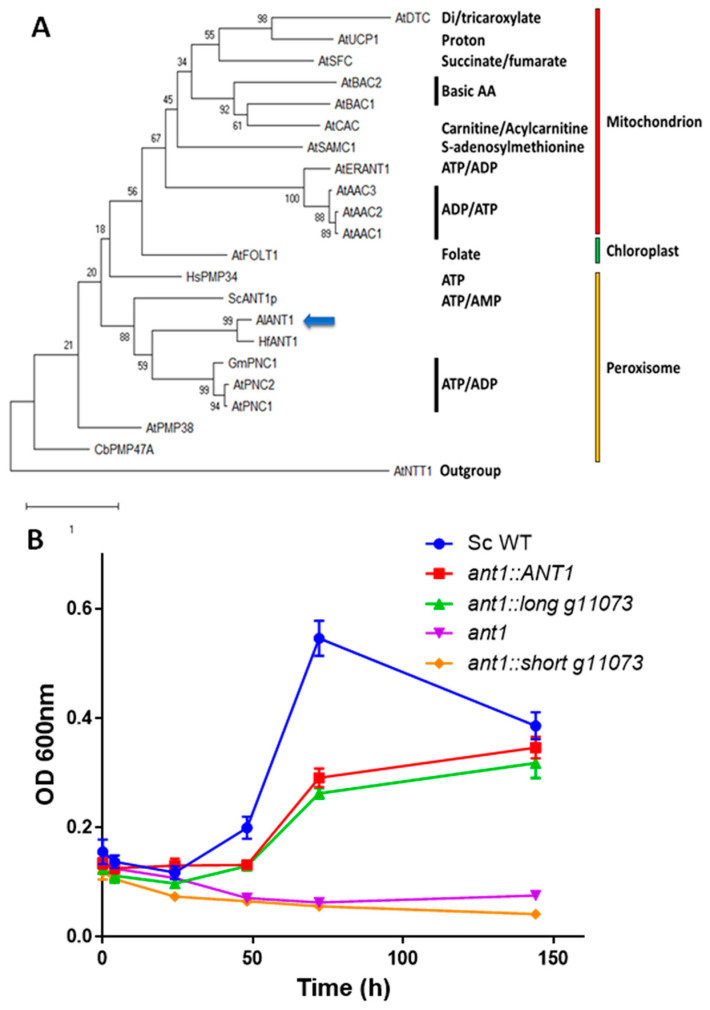
Functional analyses of g11073. (**A**) Maximum likelihood phylogenetic tree of *A. thaliana* mitochondrial carrier family proteins aligned with *A. limacinum* and *H. fermentalgiana* putative ANT1 proteins. The bootstrap values (2000 pseudoreplicates) are reported at the nodes. The analysis involved 22 amino acid sequences and 255 positions. All positions with less than 95% site coverage were eliminated (partial deletion option). (**B**) Yeast *Δant1* (in purple) was complemented either with its native *ANT1* gene (in red) or with *A. limacinum* full sequence g11073 (in green) or with *A. limacinum* short sequence g11073 (in orange). Then, growth was measured in the presence of C12:0 as a carbon source. Neither *Δant1* nor the complemented *Δant1* with the truncated g11073 could grow in the presence of C12:0. The other complemented strains could grow in the presence of C12:0, although at a lower rate than Sc WT (in blue), demonstrating that g11073 is homologous to ANT1. Each value is the mean of three biological replicates, and error bars indicate the standard deviation. Graphs and statistics were performed with GraphPad Prism^®^ software.

The g11073-translated product consisted of 316 amino acids with a theoretical molecular weight of ~35 KD. It contains the conserved solute carrier repeat profile (Solcar Prosite accession: PS50920), shaded in gray in Supplementary Figure S2. The protein sequence was analyzed using DeepLoc2 [50] and it was predicted to be a membrane-bound (likelihood 0.98) peroxisomal (likelihood 0.89) protein. To infer a possible role of g11073 as *AlANT1*, the protein sequence and that of the closely related species *Hondaea fermentalgiana* [11] (strain FCC1311 locus 007482) were aligned with protein sequences in the data set published by Arai et al., 2008 [41] and a maximum likelihood (ML) phylogenetic analysis was performed (Figure 6A). Both the putative AlANT1 and HfANT1 grouped with the PEROXISOMAL ADENINE NUCLEOTIDE CARRIER proteins (PNCs) from *Arabidopsis thaliana* (AtPNC1 and AtPNC2) and *Glycine max* (GmPNC), with ML bootstrap value of 59. The *Saccharomyces cerevisiae* ANT1 protein is found at a basal position of this group (ML 88).

Then, we aimed to confirm the physiological function of g11073. All our attempts to obtain genetically modified strains of *A. limacinum* CCAP 4062/1 failed, and, thus, a yeast complementation approach was used to validate the function of g11073. The yeast *Δant1* strain devoid of peroxisomal adenylate carrier was complemented with a YEplac195 plasmid containing either the *ANT1* gene, the complete g11073 gene, or the truncated one. The different strains displayed similar growth rates when grown in a glucose-containing medium (Supplementary Figure S3A). However, only ScANT1 and full-length g11073 proteins were detected by Western blot in protein extracts from the different complemented strains (Supplementary Figure S3B). No truncated protein was observed in any of the corresponding clones. This suggests that the truncated gene was not translated or the truncated protein was quickly degraded. As shown in Figure 6B, *Δant1* mutant cannot grow in the presence of C12:0, in agreement with previous work [45], whereas complementation with the native *ANT1* gene or the complete g11073 gene reversed the *Δant1* growth phenotype. This confirmed a peroxisomal adenylate transporter role for g11073, which was subsequently named AlANT1. The short version of g11073 was unable to complement the *Δant1* mutant, as expected.

## 4. Discussion

After a biolistic treatment aimed to produce zeocin-resistant mutants, we unexpectedly selected a clone displaying a strong lipid phenotype. Metabolic analyses pointed out a significant lipid accumulation resulting from a deficit in FA catabolism. Surprisingly, the whole genome and transcriptome sequencing did not identify any recombinant DNA, indicating that the phenotype was not the result of genetic material transfer. Rather, it likely resulted from the biolistic method itself, the particle projections provoking some DNA damages and rearrangements in nuclei [32]. Expression analyses of genes directly involved in lipid metabolism could not corroborate our observations either. In particular, the genes involved in the mobilization, transport, and oxidation of FAs were not significantly downregulated in the mutant, as one would have expected if they were to be responsible for the lower FA catabolism. However, a genomic search for structural variants identified a sizeable deletion in one of the two alleles coding for a putative peroxisomal adenylate transporter. Phylogenetic analyses and yeast complementation experiments demonstrated that the affected gene was indeed a peroxisomal adenylate carrier.

An impaired ability to import ATP into peroxisomes upholds the observed phenotype. Indeed, the catabolism of FAs in peroxisomes involves several steps. First, FAs must be transferred from the cytosolic compartment to the peroxisomes. Two independent pathways are likely involved to transport FAs across peroxisomal membranes. The first pathway transports acyl-CoA chains through an ATP Binding Cassette (ABC) transporter, such as Pxa1p/Pxa2p in yeast or CTS in plants [51,52]. Although acyl-CoA chains may enter yeast peroxisomes directly as CoA esters, it is also possible that the CoA moiety is released during the transfer, either into the cytosol or into the peroxisomal lumen [48,51,53]. CoA release prevails in plants [49,52]. Once inside the peroxisomal lumen, the corresponding free fatty acids (FFAs) are activated as CoA-esters before entering the β-oxidation cycle. This reaction is catalyzed by a peroxisomal acyl-CoA synthetase (Faa2p in yeast). A second pathway involves an unidentified transporter able to import short to medium FFA chains [45,48,52]. Once in the peroxisome, the short to medium FFAs are activated as acyl-CoAs. Peroxisomal acyl-CoA synthetases catalyze ATP-dependent reactions and require the import of ATP from the cytosol. The peroxisomal adenylate carrier [PNC in *Arabidopsis thaliana*, ANT1 (YPR128c) in *Saccharomyces cerevisiae*] belongs to the Mitochondrial Carrier Family and catalyzes the import of ATP into peroxisomes in a strict counter exchange with AMP or ADP [46,49]. In *Saccharomyces cerevisiae*, which does not accumulate lipids, a mutant devoid of peroxisomal ATP carrier (*ΔScant1*) displayed an impaired growth when C12:0 was the unique source of carbon, whereas normal growth was observed with longer chains (C18:0) [45]. In the oleaginous yeast *Yarrowia lipolytica*, a *ΔYlant1* displayed a 20% increase of total FAs, whereas a mutant *ΔYlant1ΔYlpxa1ΔYlpxa2* accumulated twice as many FAs as the WT [48]. This indicates that both FFAs and acyl-CoA are likely to enter yeast peroxisomes. In *Arabidopsis thaliana*, suppressing the ATP import into the peroxisomes strongly impaired the breakdown of storage oil. Consequently, the total level of FAs increased 10-fold in the seedlings, and oil bodies accumulated. Mutant plants were defective in seedling growth and development, unless in the presence of sucrose [49]. This illustrates the preponderant role of the peroxisomal adenylate carrier in plant β-oxidation and suggests that mainly FFAs enter plant peroxisomes.

**Figure 7:**
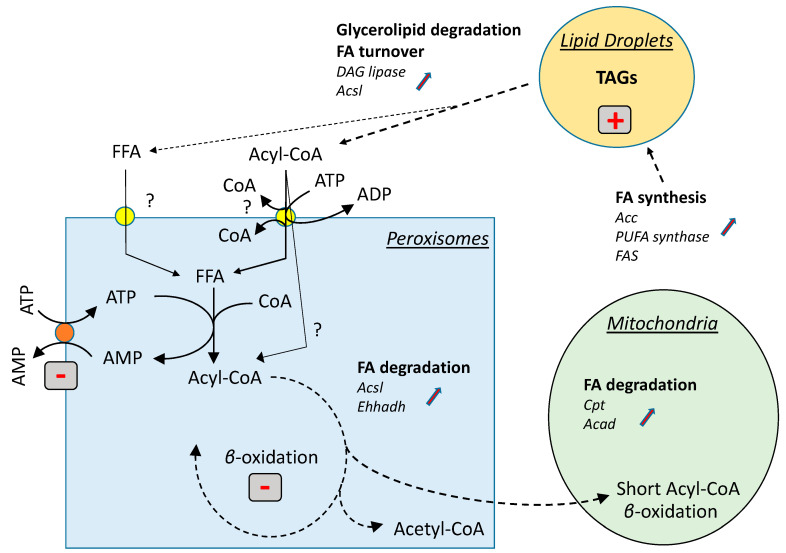
Scheme illustrating the cascade of events likely associated with a decrease of ATP import into peroxisomes. FAs produced from the degradation of TAGs enter peroxisomes through an ABC carrier or an uncharacterized free FA (FFA) transporter. Regarding the ABC carrier, it is not clear whether the CoA moiety is released in the cytosol or in the peroxisomal lumen, and if some acyl-CoAs can be transported as such. In peroxisomes, FFA are converted to acyl-CoAs, consuming ATP, before being oxidized through the β-oxidation cycle. The products of this oxidation cycle are shuttled to mitochondria for a complete oxidation to CO_2_ and H_2_O. A deficiency in ATP import into peroxisomes slows down the β-oxidation activity, resulting in lower degradation of TAGs, which accumulate. As a consequence, genes involved in FA and glycerolipid degradation are upregulated, together with genes involved in FA biosynthesis (arrows). Acsl: acyl-CoA synthase/ligase; Acc: acetyl-CoA carboxylase; Cpt: carnitine palmitoyl transferase; Acad: acyl-CoA dehydrogenase; Ehhadh: peroxisomal bifunctional enzyme of the β-oxidation; FAS: fatty acid synthase; PUFA synthase: polyunsaturated fatty acid synthase.

We did not succeed so far to stably transform *A. limacinum* strain CCAP 4062/1, thus reverse genetics’ strategies could not be used to demonstrate that the lipid phenotype described here resulted solely from a peroxisomal deficiency in ATP import. However, a number of pieces of evidence pinpointed the peroxisomal adenylate carrier as a very likely candidate. First, one of the two alleles coding for AlANT1 was seriously damaged during the biolistic treatment. The mutant displayed a lower expression of *AlANT1*, suggesting that the truncated gene was not or only minimally expressed. In addition, if translated, the truncated protein would lack two out of the three transmembrane domains, and would, therefore, most likely be inactive. Noteworthy, the truncated gene expressed in *S. cerevisiae* did not produce any truncated protein (Supplementary Figure S3), suggesting that such a protein was either not translated or rapidly degraded. Second, the lipid phenotype in LAS was associated with a lower FA catabolism activity. This phenotype is similar to those described for plant *pnc* and yeast *ant1* mutants. In response to this metabolic event, several compensatory mechanisms could be observed. In particular, genes involved in TAG degradation and acyl-CoA production in the cytosol (DAG lipase, acyl-CoA synthetase/ligase), genes for peroxisomal β-oxidation (acyl-CoA synthetase/ligase, the bifunctional enzyme Ehhadh), and those for mitochondrial β-oxidation (carnitine palmitoyltransferase, acyl-CoA dehydrogenase) were upregulated. More surprisingly, the genes involved in FA synthesis (acetyl-CoA carboxylase, FAS, PUFA synthase) were also upregulated. We have no clear explanation for this, but it is possible that the decrease in FA catabolism is perceived as a deficit in FA availability. The upregulation of lipid synthesis genes could contribute, together with the decrease in AlANT1 activity, to the large FA accumulation. These potential metabolic events are summarized in Figure 7.

Interestingly, the C16:0/C22:6 ratio fluctuated along the growth in both strains. In WT, C16:0 decreased faster than C22:6 during the rapid oil breakdown phase, whereas it increased faster than C22:6 in the oil accumulation phase. This could reflect a faster dynamics/turnover for C16:0 than for C22:6, with C16:0 being more rapidly mobilized/oxidized than 22:6, and more rapidly synthesized by the FAS system than C22:6 by the PUFA synthase complex. A higher proportion of C16:0 was also observed in LAS (35% of total FAs) compared with WT (20% of total FAs). In LAS, the genes encoding FAS and PUFA synthase were similarly upregulated (log_2_FC = 1.3 and 1.4–2, respectively), and it is difficult to estimate whether or not these regulations can support the observed change in the C16:0/C22:6 ratio. However, it is also possible that a sorting in the import of FAs into peroxisomes exists in *A. limacinum*, as it does in *S. cerevisiae*. If this is true, more C16:0 than C22:6 would enter peroxisomes as FFAs, and, thus, the β-oxidation of C16:0 in peroxisomes could be more ATP dependent than C22:6.

Biolistic transformation is a convenient method, well adapted to organisms having thick cell walls such as plants [54,55] or silica shells such as diatoms [30]. However, as already reported [32,33,56], it must be kept in mind that interpretation of a biolistic transformation needs caution. The results presented here appear relatively simple since no transgenic material was transferred into the genome of *A. limacinum*. In this particular case, the biolistic experiment can be seen as a random mutagenesis experiment, possibly enhanced by the action of the selection agent, the zeocin, which induces double-strand breaks [57]. In cases where the transfer of genetic material is effective, it may be difficult to determine, without sequencing the entire genome, what is due to transformation itself and what is due to collateral damages. The parallel study of independent, transformed lines is a pragmatic way to address this technical issue. Sequence breakage and reassembly are common events with biolistic transformation [56,58]. DNA repair involves mechanisms such as non-homologous end joining (NHEJ) or homology directed repair (HDR), eventually leading either to simple insertion with no other evidence of genome damage, chromothripsis-like rearrangements, or genomic deletions [32]. It is also possible that the specific nature of *A. limacinum* CCAP 4062/1 contributes to increase the probability to get genomic impairment events during biolistic treatments. Indeed, several cell types characterize the *A. limacinum* life cycle, including mononucleated and multinucleated cells [6,35]. The presence of cells with several nuclei might increase the complexity of events resulting from the transformation process and the bombardment procedure.

## 5. Conclusions

In conclusion, we selected by serendipity a mutant of *A. limacinum* presenting a large deletion in one of the two alleles coding for a peroxisomal adenylate carrier. This deletion resulted from biolistic damage. The large lipid increase associated with this event and the inheritability of this phenotype strongly support the hypothesis that the peroxisomal adenylate carrier is a very promising target to engineer the lipid content in Thraustochytrids.

## Figures and Tables

**Table 1:** Differentially expressed genes involved in FA synthesis and FA degradation (LAS versus WT, log_2_FC > |1|). Genes and predicted functions referred to the Aurli1 database for *Aurantiochytrium limacinum* ATCC MYA-1381.

Name	Symbol	Reference in Aurli1	Log_2_FC
**Fatty Acid Synthesis**			
Acetyl-CoA carboxylase	ACC	gw1.10.632.1	1.45
PUFA synthase	PufaA	fgenesh1_pg.14_251	2.21
PUFA synthase	PufaB	estExt_fgenesh1_kg.C_140136	1.6
PUFA synthase	PufaC	estExt_fgenesh1_kg.C_190026	1.38
Fatty Acid Synthase I	Fas1	e_gw1.21.366.1	1.33
**Fatty Acid Degradation**			
Mitochondrial carnitine/acylcarnitine transporter	CACT	fgenesh1_kg.16_67_isotig06873	1.78
carnitine palmitoyl transferase CPT1	Cpt1	fgenesh1_kg.6_177_isotig02684	1.28
carnitine palmitoyl transferase CPT3	Cpt2-2	estExt_fgenesh1_kg.C_60140	1.37
Acyl-CoA dehydrogenase	Acad4	estExt_Genewise1.C_9_t10269	1.17
Enoyl-CoA hydratase	Ech6	gm1.2779_g	1.58
Bifunctional enzyme	Ehhadh3	gm1.11390_g	1.17
Long chain acyl-CoA synthase/ligase	Acsl	e_gw1.11.199.1	3.01
Long chain acyl-CoA synthase/ligase	Acsl	e_gw1.2.456.1	2.93
Long chain acyl-CoA synthase/ligase	Acsl	estExt_fgenesh1_kg.C_40024	2.78
Long chain acyl-CoA synthase/ligase (peroxisomal)	Acsl	estExt_fgenesh1_kg.C_160013	2.51
Long chain acyl-CoA synthase/ligase (peroxisomal)	Acsl	estExt_fgenesh1_kg.C_160012	2.07
Long chain acyl-CoA synthase/ligase	Acsl	gw1.11.174.1	2.06
Long chain acyl-CoA synthase/ligase	Acsl	fgenesh1_pm.5_56	1.51
Long chain acyl-CoA synthase/ligase (peroxisomal)	Acsl	e_gw1.16.213.1	1.31
**Glycerolipid Degradation**			
DAG lipase	Dagl6	fgenesh1_pg.9_214	2.46
DAG lipase	Dagl8	gm1.4322_g	1.08

**Figure A1:**
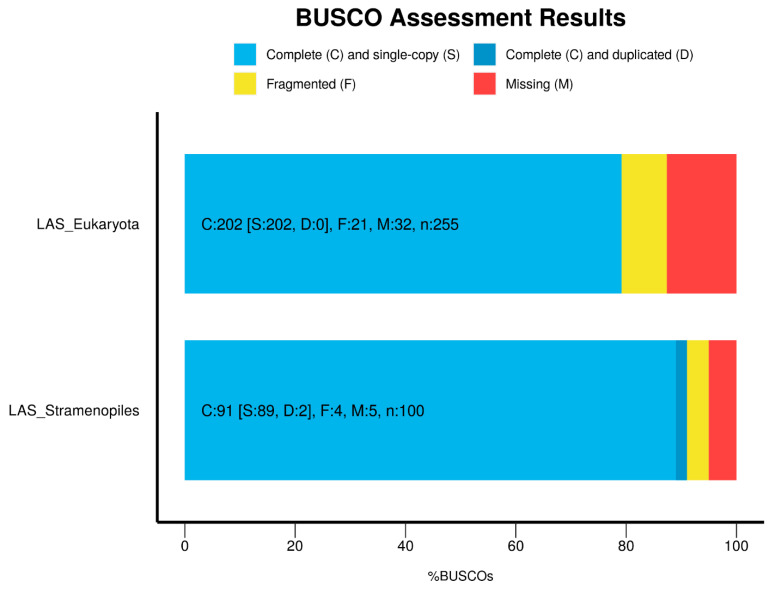
Results of the BUSCO analysis highlighting the presence of complete and single copy Eukaryotic and Stramenopiles genes in the assembly. Letters indicate the BUSCO categories presented in the figure, numbers indicate the number of genes composing a category. ‘n’ indicates the total number of genes in all BUSCO categories.

**Figure A2:**
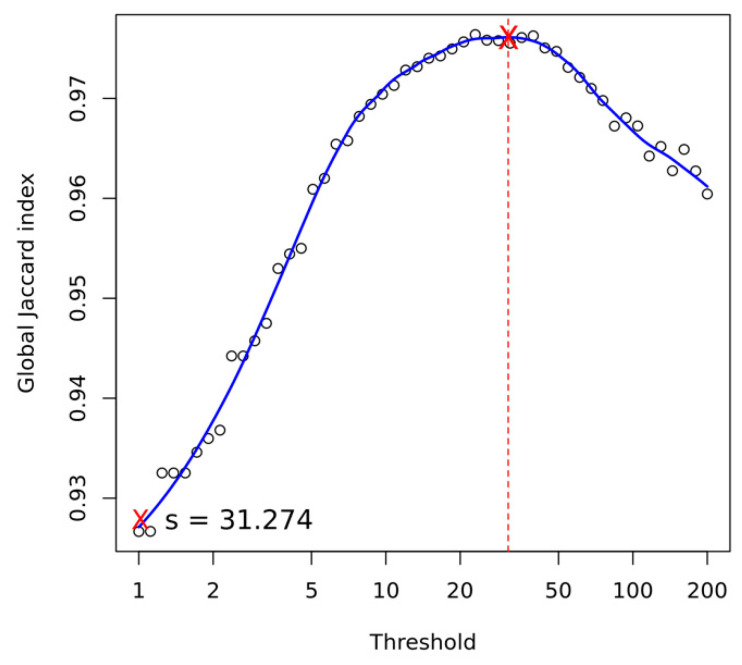
Results of the low expression filtering step performed with HTSFilter. The algorithm calculated a Global Jaccard index (blue line) of similarity (on the *Y*-axis) between the samples in function of different minimum normalized read counts (on the *X*-axis, red crosses indicate the minimum and the maximum values). The graphic shows that for s = 31.274 the replicates have the highest similarity; thus, this value was used as a threshold.

**Figure A3:**
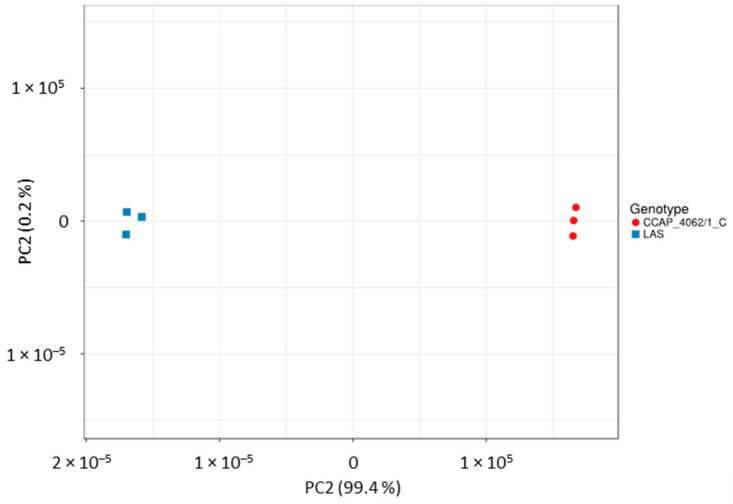
Principal Component Analysis showing the clustering of the RNA-seq samples (red circles = wild type CCAP 4062/1, blue squares = LAS mutant) using the TMM normalized expression values. The percentage of explained variance is indicated in parenthesis. LAS and CCAP_4062/1 replicates are indicated as blue squares and red circles, respectively.

## Data Availability

All data relative to this study are presented in this article and in the Supplementary material. Sequencing data are available at https://www.ncbi.nlm.nih.gov/bioproject/PRJNA728408/.

## Supplementary Materials

The following are available online at https://www.mdpi.com/article/10.3390/cells10102680/s1, Figure S1: Growth curves of the wild type (WT) and lipid accumulating (LAS) strains in R medium. Figure S2: Alignment of amino acid sequences from *Aurantiochytrium limacinum* ANT1 (bottom line) with *Arabidopsis thaliana* PNC1 and PNC2, *Glycine max* PNC1, and *Saccharomyces cerevisiae* ANT1. Shaded in gray, the solute carrier (solcar) repeat motifs. Figure S3: Complemented strains of *Saccharomyces cerevisiae Δant1*. All the plasmids incorporated in the different strains were extracted and sequenced for validation. (A) growth curves of WT, *Δant1,* and the three *Δant1* complemented strains in the presence of glucose. (B) Western blot showing the presence of the complemented proteins in protein extracts from different clones of the three complemented strains (three independent clones per construct). For *Δant1* complemented with the truncated form of g11073, none of the selected clones expressed the truncated g11073 protein. Table S1: List of DE genes involved in lipid metabolism. Table S2: List and main characteristics of the structural variants.

## Appendix A

### Appendix A.1. Genomic and Transcriptomic Approach

Here we report the genome sequencing, assembling and annotation of a transgenic line isolated by serendipity after biolistic genetic transformation with a zeocin expression plasmid of the parent *Aurantiochytrium limacinum* strain CCAP_4062/1. The strain turned out to be a false positive because it did not contain any trace of the plasmid bombarded on the cells. Nevertheless, the mutant showed a strong lipid accumulation phenotype (henceforth referred to as Lipid Accumulator Strain, LAS). In previous works, we demonstrated that the parent strain we used to produce LAS mutant belongs to *Aurantiochytrium limacinum* species [11] and that its genome [36] shows a high degree of collinearity and an Average Nucleotide Identity (ANI) of 98.89% with *A. limacinum* ATCC^®^ MYA1381^TM^ reference genome (https://phycocosm.jgi.doe.gov/Aurli1/Aurli1.info.html, accessed on 1 April 2020). Noteworthy, *A. limacinum* ATCC^®^ MYA1381^TM^ is the type species of the genus *Aurantiochytrium* (as strain SR21) [59].

### Appendix A.2. Genome Assembly

The raw sequencing dataset included long reads produced with PacBio Sequel I (Table A1). About 676,000 polymerase reads with an N50 of 15,440 bp and a total of about 6 Gbp were produced. Considering that the estimated genome size of *A. limacinum* is about 60 Mbp, the PacBio data corresponded to an estimated 97× coverage.

**Table A1:** Description of the raw DNA sequencing dataset used in this study.

	PacBio Sequel I
Sequenced Bases	5,967,660,711 bp
Number of Reads	676,120
Sequencing Layout	Single End Long Reads
Max Read Length	70,183 bp
Read N50	15,440 bp
Estimate Genome Coverage	97×

PacBio subreads were used to generate a complete LAS genome assembly. For this purpose, the reads were first corrected applying three iterations of the self-correction algorithm MECAT [60] with three k-mers length. A draft assembly was then generated with the software Canu [61]. In order to correct residual sequencing errors, RNA-seq data from three LAS samples were used in conjunction with the Pilon algorithm [62] to perform four rounds of corrections. A total of 33,351 corrections were applied, 94% of which were performed during the first iteration.

The final LAS assembly included 147 scaffolds with a total size of 59.28 Mbp (Table A2). The assembly showed high contiguity having an N50 of 1.2 Mbp and an L50 of 16 scaffolds. About 98.69% of the PacBio corrected reads could be mapped back to the assembly. The BUSCO [63] analysis showed that 79% and 91% of Eukaryotic and Stramenopile conserved single copy genes were detected in the assembly (Figure A1).

**Table A2:** Genome assembly statistics.

	*Aurantiochytrium limacinum* LAS
Number of Scaffolds	147
Genome Size	59,284,731 bp
Number of Contigs larger than 50 Kbp	106
N50	1,222,801 bp
L50	16
Largest Contig	3,306,648 bp
GC Content	45.28%

### Appendix A.3. RNA Sequencing

Six RNA samples were sent out for sequencing, three from the *A. limacinum* LAS and three for *A. limacinum* CCAP_4062/1. Prior to further analysis, a quality check was performed on the raw sequencing data, removing low quality portions (Q < 25) while preserving the longest high-quality part of NGS reads. A total of 211.6 and 189.9 million of raw and trimmed reads were produced (Table A3).

**Table A3:** Number of reads before and after quality check.

Sample ID	Raw Reads	Trimmed Reads
CCAP_4062/1_A	43,636,868	39,160,710
CCAP_4062/1_B	28,851,896	25,815,902
CCAP_4062/1_C	33,658,698	29,801,748
LAS_4A	32,324,384	28,722,048
LAS_4B	31,644,014	28,589,096
LAS_4C	41,736,058	38,014,092

On average 94.5% of the reads could be mapped on the reference genome of *A. limacinum* ATCC^®^ MYA1381^TM^ with STAR aligner [64] (Table A4). Most of the reads mapped uniquely on the genome.

**Table A4:** Results of the RNA-seq data mapping against the reference genome.

Sample ID	Uniquely Mapped (%)	Multi-Mapped (%)
CCAP_4062/1_A	91.05%	2.06%
CCAP_4062/1_B	91.24%	2.02%
CCAP_4062/1_C	90.81%	2.14%
LAS_4A	94.23%	1.76%
LAS_4B	93.58%	1.80%
LAS_4C	94.06%	1.77%

Gene expression quantification and the expression normalization were performed in order to carry out a differential expression analysis between LAS and CCAP_4062/1. First, lowly expressed genes were filtered out using the HTSFilter algorithm (Figure A2) [65] which produced a dataset of about 10,000 genes to be further analysed.

A Principal Component Analysis (PCA) using the normalised gene expression values as input was carried out in order to estimate the quality of the experiment in terms of reproducibility of the replicates (Figure A3). The WT replicates group together and well differentiated along the principal component 1 (amount of variance explained 99.39%) from the group of LAS replicates.

About 7300 genes were found to be significantly differentially expressed between LAS and CCAP_4062/1, 1974 of which with an absolute logarithmic fold change higher than 1.

### Appendix A.4. Structural Variant Calling

In order to identify all the possible structural variations between LAS and its parent CCAP_4062/1, a structural variant calling was performed. As a first step the PacBio reads of LAS and CCAP_4062/1 were mapped against the reference genome of CCAP_4062/1 [36]. The statistics of the mapping for the two samples are reported in Table A5.

**Table A5:** Statistics of mapping LAS and CCAP_4062/1 PacBio long reads against the CCAP_4062/1 genome [36].

Statistics	LAS	CCAP_4062/1
Percentage of Mapping	99.66%	90.76%
Average Mapping Quality	47.73	50.33
Average Coverage	30	92

The software SVIM [66] was used to perform the structural variation (SV) analysis. Small variants, such as SNPs and small InDels (up to 40 bp) were not considered with this approach. The CCAP_4062/1 reads were also analysed to identify background variants that are not due to true biological variation but to the noise of the sequencing technology. By using this approach, the LAS variants shared with CCAP_4062/1 were filtered out as false positives. About 6400 variants were found in the two samples (Supplementary Table S1), but only 13 were specific for LAS.

### Appendix A.5. Experimental Design, Materials, and Methods

#### Appendix A.5.1. The Strains and Maintenance Culture Conditions

*Aurantiochytrium limacinum* Lipid Accumulator Strain (LAS) was obtained by serendipity as a false positive after nanoparticle bombardment of the *A. limacinum* strain CCAP_4062/1. The LAS is stored at −80 °C as glycerol stock at the Laboratory of Plant and Cell Physiology (Grenoble, France). Cultures were maintained on both liquid R medium and 1% agar-R Petri-dish. The R medium was produced with 50% *(v/v)* ESAW medium containing 6% *(w/v)* glucose as source of carbon and 2% (*w/v*) yeast extract (the full recipe is available in Supplemental Table S1 in reference [61]). Liquid cultures were produced by inoculating 50 mL fresh R medium at 5 × 10^5^ cells·mL^−1^ in sterile Pyrex^®^ Erlen-Meyer 250 mL flasks and incubated at 20 °C and 100 r.p.m. in a Multitron HT Infors shaker incubator.

#### Appendix A.5.2. Cloning and Genetic Transformation

The zeocin resistance gene (*Sh Ble*) was cloned under the polyubiquitin promoter/terminator. An ORF encoding a yeast UBI4 polyubiquitin homologous gene was identified in the genome of CCAP 4062/1 and a 917 pb sequence upstream the ORF was amplified using the primers PromUbi2SacI-F 5′-TTGAGCTCAGAGCGCGAAAGAGAGTGCCGGAATTC-3′; PromUbi2BamHI-R-5′GCGGATCCGAAATTGACCTTACTGCCTCTCCTGTG-3′ to add the restriction sites SacI in 5′and BamHI in 3′ of the sequence. A 935 pb sequence downstream the ORF was amplified with the primers TermUbi2SphI-F 5′-GGGCATGCTGTCAAAACCGGGGTTAGTGACATTGA-3′; TermUbi2HindIII-R 5′-GGAAGCTTCCATTTGCCTCTGCGTGAAATTCAATC-3′ to add the restriction sites SphI in 5′ and HindIII in 3′ of the sequence. A 375 pb sequence encoding *Sh Ble* from the commercial plasmid pTEF1 was amplified with the primers ZeoS1BamHI 5′-GCGGATCCATGGCCAAGTTGACCAGTGCCGTTCC-3′ and ZeoS1SalI 5′-GCGTCGACTCAGTCCTGCTCCTCGGCCACGAAGT-3′ to add the restriction sites BamHI in 5′ and SalI in 3′ of the sequence. The fragments were sequentially inserted into the multiple cloning site of the pUC19 plasmid to obtain the 4798 bp plasmid pUbi-Zeo.

Biolistic transformation was carried out on 2 × 10^7^ cells in exponential growth plated on 1% agar-R Petri-dish. Tungsten particles (BioRad Tungsten M-17 Microcarriers) were coated with 1 µg plasmid DNA via CaCl_2_ and spermidine and projected onto cells via a Biolistic PDS-1000/He system (BioRad) using 1550 psi rupture disks (BioRad) following the manufacturer instructions.

#### Appendix A.5.3. Nucleic Acid Extraction and Sequencing

DNA was extracted from a six-day old LAS culture by phenol:chloroform:isoamyl alcohol (25:24:1) on lysed cells and precipitated with Na-Acetate 3M pH 5 and absolute ethanol. RNA was extracted from both the LAS and the CCAP_4062/1 strains after 48 h of cultivation at the same conditions as described above, i.e., when cultures were at the end of the exponential phase of growth [35]. RNA was extracted from 1.5 × 10^7^ cells using a TRI reagent (Sigma Aldrich) protocol as described in reference [26]. Quality and concentration of the extracted nucleic acids were estimated using a Thermo Scientific™ NanoDrop 2000 Spectrophotometer and Qubit Flex Fluorometer. RNA integrity was assessed by electrophoresis (2% agarose Tris base-acetic acid-EDTA (TAE) buffer). Extracted nucleic acids were sent out to Macrogen (Seoul, Korea) for sequencing. DNA was prepared according to a guide for preparing SMRTbell template for sequencing on the PacBio Sequel System. The templates were sequenced using SMRT Sequencing. Libraries for RNA sequencing were produced and processed following the manufacturer’s instructions and sequenced on paired-end 125 bp mode on HiSeq2500 (Illumina, San Diego, CA, USA). The CASAVA 1.8.2 version of the Illumina pipeline was used to process raw data for both format conversion and de-multiplexing.

### Appendix A.6. Bioinformatics Methods

#### Appendix A.6.1. Genome Assembly

PacBio Sequel I subreads were processed with three iterations of MECAT v1.0 with k-mer lengths of 19, 31, and 41 nucleotides to obtain self-corrected reads which were then used with Canu v2.1.1 with default parameters. Assembly correction was performed using the Illumina RNA-seq reads which were first trimmed using the software BBDuk [67] setting a minimum base quality of 25 and a minimum length of 35 nt. After trimming, the reads were mapped against the assembly with STAR v2.7.3a in double-pass mode and the obtained BAM files were processed with Opossum v0.2 [68] to split intron spanning reads. Finally, the obtained BAM files were used with Pilon v1.23 with the options “—fix all –minmq 30 –minqual 30”, four iterations were performed to obtain the final genome assembly. Genome assembly statistics were produced with the software QUAST v4.0 [69] whereas BUSCO analyses were performed with the software version 4 and the eukaryote_odb10 and stramenopiles_odb10 datasets.

For genome structural annotation, a previously generated Augustus model was used [36]. Gene functional annotations were obtained using the software PANNZER2 [70] with the following options: Minimum query coverage 0.4 or minimum subject coverage 0.4, and Minimum alignment length 50. The ARGOT [71] scoring function implemented in PANNZER2 as default advanced option for Gene Ontology Annotation that proved to be the best [70], was chosen.

#### Appendix A.6.2. RNA-seq Analysis

The high-quality reads obtained as described above were mapped against the *A. limacinum* ATCC^®^ MYA1381^TM^ reference genome with STAR aligner (version 2.5.2b). FeatureCounts (version 1.5.1) [72] was used to calculate gene expression values as raw fragment counts. Normalization was applied to the raw fragment counts by using the Trimmed Mean of M-values (TMM) normalization and Fragments Per Kilobase Million (FPKM) normalization. The HTSFilter v3.13 package was used to remove the genes either showing no expression or too much variability for each comparison. HTSFilter package implements a filtering procedure for replicated transcriptome sequencing data based on a Jaccard similarity index [73]. Differential expression analysis was performed with the edgeR algorithm on the TMM normalized data and selecting the genes with an FDR ≤ 0.05.

#### Appendix A.6.3. Structural Variant Calling

LAS and CCAP_4062/1 corrected PacBio reads were mapped against the CCAP_4062/1 reference genome [36] using the NGMLR mapper with default parameters. Quality of mapping was evaluated with Qualimap. Structural Variant calling v2.2.1 was then performed with the SVIM algorithm using LAS and CCAP_4062/1 files. VCF files were converted to BED format with in-house scripts and then bedtools intersect was used to extract private LAS structural variants.
