# Supplementary material for: An Oil Hyper-Accumulator Mutant Highlights Peroxisomal ATP Import as a Regulatory Step for Fatty Acid Metabolism in Aurantiochytrium limacinum
Source: Cells. 2021 Oct 6;10(10):2680. doi: 10.3390/cells10102680 (PMC8534400; doi:10.3390/cells10102680)
Supplement: Supplementary file 1 [file cells-10-02680-s001.zip › Supplementary data and methods_MDPI.pdf]

Supplementary data and methods

Supplementary Methods

Yeast transformation

<u>Primers for *Saccharomyces cerevisiae* transformations</u>

Name	Description	Sequence 5'-3'
FR4	Promoter PMA1 fw	AATTTCACACAGGAAACAGCTATGACCATGATTACGCCAAGCTTGCATGCGCGGCACTTCCAGGCCTCG
FR5	Promoter PMA1 rv	AATGCAGACTCTAGAGTTAACATATTGATATTGTTTGATAATTAAATCTTTCTTATCTTC
FR6	ANT1 Sc fw	TAAGAAAGATTTAATTATCAAACAATATCAATATGTTAACTCTAGAGTCTGCATTAACTG
FR7	ANT1 Sc rv	ATACGGATAGCCCGCATAGTCAGGAACATCGTATGGGTAAGTGGAAGCCAGCTTGCGTTG
FR8	HA-tADH1 Fw	TAAAGCACAACGGACAACGCAAGCTGGCTTCCACTTACCCATACGATGTTCCTGACTATG
FR9	HA-tADH1 rv	CGTTGTAAAACGACGGCCAGTGAATTCGAGCTCGGTACCCGGCCGGTAGAGGTGTGGTC
FR12	Promoter PMA1 rv	AGCTTCGAGCCCAGAATCTCCATATTGATATTGTTTGATAATTAAATCTTTCTTATCTTC
FR13	ANT1 long Al Fw	GAAGATAAGAAAGATTTAATTATCAAACAATATCAATATGGAGATTCTGGGCTCGAAGC
FR14	ANT1 long/short Al rv	ATACGGATAGCCCGCATAGTCAGGAACATCGTATGGGTACTTCACCTGCTTTTGCGAGC
FR15	HA-tADH1 Fw	AAGCTTCCATCATGGGCTCGCAAAAGCAGGTGAAGTACCCATACGATGTTCCTGACTATG
FR16	Promoter PMA1 rv	CGAGTGGTGATAATACGAGTATTATTGATATTGTTTGATAATTAAATCTTTCTTATCTTC
FR17	ANT1 short Al Fw	AAGATAAGAAAGATTTAATTATCAAACAATATCAATAATACTCGTATTATCACCACTCGC

<u>In vivo cloning strategy to construct the plasmids expressing the gene of interest</u>

1-ANT1 ***Saccharomyces cerevisiae***
PCR1: FR4-FR5, 700 pb; PCR2: FR6-FR7, 984 pb; PCR3: FR8-FR9, 310 pb.
In vivo cloning: YEplac195 digested PstI/BamHI + PCR1 + PCR2 + PCR3
2-ANT1-long ***Aurantiochytrium limacinum***
PCR4: FR4-FR12, 700 pb; PCR5: FR13-FR14, 948 pb ; PCR6: FR15-FR9, 310 pb.
In vivo cloning: YEplac195 digested PstI/BamHI + PCR4 + PCR5 + PCR6
3-ANT1-short ***Aurantiochytrium limacinum***
PCR7: FR4-FR16, 700 pb; PCR8: FR17-FR14, 582 pb ; PCR6: FR15-FR9, 310 pb.
In vivo cloning: YEplac195 digested PstI/BamHI + PCR7 + PCR8 + PCR6

Primers for qRT-PCR analyses of *AlANT1* in LAS and WT

List of primers used for qRT-PCR (Fig. 5B). The reference genes are reported in bold.

Gene ID	Annotation	Forward Primer	Reverse Primer	Efficiency
e_gw1.9.603.1	Peroxisomal adenylate carrier – Amp3	CAGACAGCTCTATGCGATCC	GCATCGTAGTGCCAAGACC	1,95
e_gw1.9.603.1	Peroxisomal adenylate carrier – Amp4	TGACGTTTCCGTTCATTCG	TCCTCAGTCGGCTCAATACC	1.94
**estExt_fgenesh1_kg.C_30063**	**Cystein desulfurase NFS1**	CGAGGGTTTTGAGGTGACAT	CACCAATTTCGTTGTTGACG	2,02

Supplementary Data

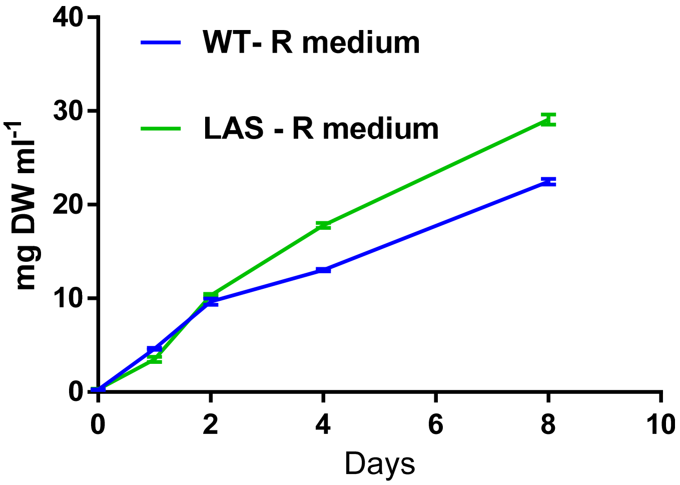

Figure S1. Growth curves of the wild type (WT) and lipid accumulating (LAS) strains in R medium.

Supplementary Table S2: List and main characteristics of the structural variants determined from PacBio sequencing (InDel ≥ 40 bp). As shown, three genes were not expressed in neither WT nor in LAS, the two remaining genes, i.e. g1125 (homozygous variant) and g11073 (heterozygous variant), were expressed.

**Homzygous variants**												
**scaffold**	**Start**	**End**	**Type of variant**	**Locus**	**Blast against reference genome Aurli1**	**Predicted**	**WT read count**			**LAS read count**		
00125	771	824	Deletion	g1111			0	0	1	0	0	0
00125	19143	19189	Insertion	g1125		PAN_1 domain containing protein	30	32	33	11	13	12
00125	60524	60576	Insertion	g1149	jgi|Aurli1|35206|fgenesh1_pg.11_#_13	Phage N-6 adenine methyltransferease	0	0	0	0	0	0
00674	607277	607338	Deletion	g7841	jgi|Aurli1|1921|gm1.1921_g	TAUE integral membrane proteins containing transmembrane helices	0	0	0	0	0	0
**Heterozygous variant**												
**scaffold**	**Start**	**End**	**Variant**	**Gene**	**Blast against reference genome Aurli1**	**Predicted**	**WT read count**			**LAS read count**		
00759	206415	207258	Deletion	g11073	jgi|Aurli1|76398|e_gw1.9.603.1	Peroxisomal adenine nucleotide carrier 1	15	16	13	11	13	10

Sequences of the putative *Aurantiochytrium limacinum* ANT1 proteins

>WT_g11073_protein

MEILGSKLALSNALSGAAADAFSSAALYPLDVVKVQMQASKTHSLYDVMKHLMEHPSEAYKGLQTKIVASVQ
QKFQYFYVYALLRQLYAIRTGQKPGALVDLVIGYLSALEGLGTTMPFEVVNTRIITTRKVHKDEKPPGFWETFNE
ILEKEGAKSFYRTLPASMILCINPAITYVVFEELKSRILERAHSGSQVLTTAQALVVGVISKSIASIVTFPFIRAKVLM
SVWKKSHDMHLEEERKRGIEPTEEELNRQTPGLIATMEAVLQNEGVLGLYKGLGATLFKGVSNAALMLAVKE
KIYVVVQASIMGSQKQVK

>LAS_g11073_protein

NTRIITTRKVHKDEKPPGFWETFNEILEKEGAKSFYRTLPAS**M**ILCINPAITYVVFEELKSRILERAHSGSQVLTTA
QALVVGVISKSIASIVTFPFIRAKVLMSVWKKSHDMHLEEERKRGIEPTEEELNRQTPGLIATMEAVLQNEGVLG
LYKGLGATLFKGVSNAALMLAVKEKIYVVVQASIMGSQKQVK

>jgi|Aurli1|76398|e_gw1.9.603.1_protein, putative ‘peroxisomal adenylate carrier’

MEHPSEAYKGLQTKIVASVQQKFQYFYVYALLRQLYAIRTGQKPGALVDLVIGYLSALEGLGTTMPFEVVNTRII
TTRKVHKDEKPPGFWETFNEILEKEGAKSFYRTLPASMILCINPAITYVVFEELKSRILERAHSGSQVLTTAQALV
VGVISKSIASIVTFPFIRAKLNRQTPGLIATMEAVLQNEGVLGLYKGLGATLFKGVSNAALMLAVKEKIYVVVQA
SIMGSQKQVK

```
              ....|....| ....|....| ....|....| ....|....| ....|....|
                  5         15         25         35         45
ScANT1p       M-----LTL  ESALTGAVAS AMANIAVYPL DLSKTIIQSQ VSPSSSEDSN
AtPNC1        MGVDL--ESV SEATSGAIGS LLSTTILYPL DTCKSKFQAE VRARG-----
AtPNC2        MGVDLDLESI SEATSGAIGS LLSTTILYPL DTCKSKFQAE IRVRG-----
GmPNC1        MNVDL--ESL AEATSGAIGS LISTTILYPL DTCKTKYQAE ARSSG-----
A1ANT1        MEILGSKLAL SNALSGAAAD AFSSAALYPL DVVKVQMQAS KTHS------

              ....|....| ....|....| ....|....| ....|....| ....|....|
                 55         65         75         85         95
ScANT1p       EGKVLPNRRY KNVVDCMINI FKEKGILGLY QGMTVTTVAT FVQNFVYFFW
AtPNC1        ------QQKY RYLSDVMWEA ISKGQVFSLY QGLGTKNFQS FISQFIYFYS
AtPNC2        ------QQKY RYLSDVFWEA ISSGNVLSLY QGLGTKNLQS FISSFIYFYS
GmPNC1        ------RTKY RNLTDVLLEA ISNRQVLSLY QGLGTKNLQS FISQFVYFYG
A1ANT1        ---------- --LYDVMKHL MEHP--SEAY KGLQTKIVAS VQQKFQYFYV

              ....|....| ....|....| ....|....| ....|....| ....|....|
                105        115        125        135        145
ScANT1p       YTFIRKSYMK HKLLGLQSLK NRDGPITPST IEELVLGVAA ASISQLFTSP
AtPNC1        YSYFKRVHSE ---------- -RTGSKSIGT KANLLIAAAA GACTSVLIQP
AtPNC2        YSYFKRLHSQ ---------- -RIGSKSIGT KANLLIAAAA GACTSVLTQP
GmPNC1        YSYFKRLYLE ---------- -KSGYKSIGT KANLVIAAAA GACTAIATQP
A1ANT1        YALLRQLYAI ---------- -RTGQK-PGA LVDLVIGYLS ALEGLGTTMP

              ....|....| ....|....| ....|....| ....|....| ....|....|
                155        165        175        185        195
ScANT1p       MAVVATRQQT VHSAESAKFT NVIKDIYREN NGD--ITAFW KGLRTGLALT
AtPNC1        LDTASSRMQT S----EFGES KGLWKTLTEG S----WADAF DGLGISLLLT
AtPNC2        LDTASSRMQT S----EFGKS KGLWKTLTDG S----WGNAF DGLGISLLLT
GmPNC1        LDTASSRMQT S----EFGKS KGLLKTLTEG N----WSDAF DGLSISLLLT
A1ANT1        FEVVNTRIIT TRKVHKDEKP PGFWETFNEI LEKEGAKSFY RTLPASMILC

              ....|....| ....|....| ....|....| ....|....| ....|....|
                205        215        225        235        245
ScANT1p       INPSITYASF QRLKEVFFHD HSNDAG---- --SLSAVQNF ILGVLSKMIS
AtPNC1        SNPAIQYTVF DQLKQHLLKQ KNAKAENGSS PVVLSAFMAF VLGAVSKSVA
AtPNC2        SNPAIQYTVF DQLKQNLLEK GKAKSNKDSS PVVLSAFMAF VLGAVSKSAA
GmPNC1        SNPAIQYTVF DQLKQRALKN KQDNADKGTS PASLSAFMAF LLGAISKSIA
A1ANT1        INPAITYVVF EELKSRILER AHSGSQ---- --VLTTAQAL VVGVISKSIA

              ....|....| ....|....| ....|....| ....|....| ....|....|
                255        265        275        285        295
ScANT1p       TLVTQPLIVA KAMLQSAGS- ---------- ---------- --KFTTFQEA
AtPNC1        TVLTYPAIRC KVMIQAADES KENETKKPRR R--------- --TRKTIPGV
AtPNC2        TVITYPAIRC KVMIQAADDS KENEAKKPRK R--------- --IRKTIPGV
GmPNC1        TCLTYPAIRC KVIIQAADS- -AEETSKTKI K--------- --SQKTVLSV
A1ANT1        SIVTFPFIRA KVLMSVWKKS HDMHLEEERK RGIEPTEEEL NRQTPGLIAT

              ....|....| ....|....| ....|....| ....|....| ....|....|
                305        315        325        335        345
ScANT1p       LLYLYKNEGL KSLWKGVLPQ LTKGVIVQGL LFAFRGELTK SLKRLIFLYS
AtPNC1        VYAIWRKEGM LGFFKGLQAQ ILKTVLSSAL LLMIKEKITA TTWILILAIR
AtPNC2        VYAIWKKEGI LGFFKGLQAQ ILKTVLSSAL LLMIKEKITA TTWILILAIR
GmPNC1        LYGIWKREGI LGYFKGLHAQ ILKTVLSSAL LLMIKEKISA STWVLILALK
A1ANT1        MEAVLQNEGV LGLYKGLGAT LFKGVSNAAL MLAVKEKIYV VVQASIMGSQ

              ....|....| ....|
                355        365
ScANT1p       SFFLKHNGQR KLAST
AtPNC1        RTLFLTNTKG KLRSP
AtPNC2        -TLFVT--KA RLKSP
GmPNC1        RYLLLP--RG KVKNL
A1ANT1        KQVK------ -----
```

Figure S2: Alignment of amino acid sequences from *Aurantiochytrium limacinum* ANT1 (bottom line) with *Arabidopsis thaliana* PNC1 and PNC2, *Glycine max* PNC1 and *Saccharomyces cerevisiae* ANT1. Shaded in grey the solute carrier (solcar) repeat motifs.

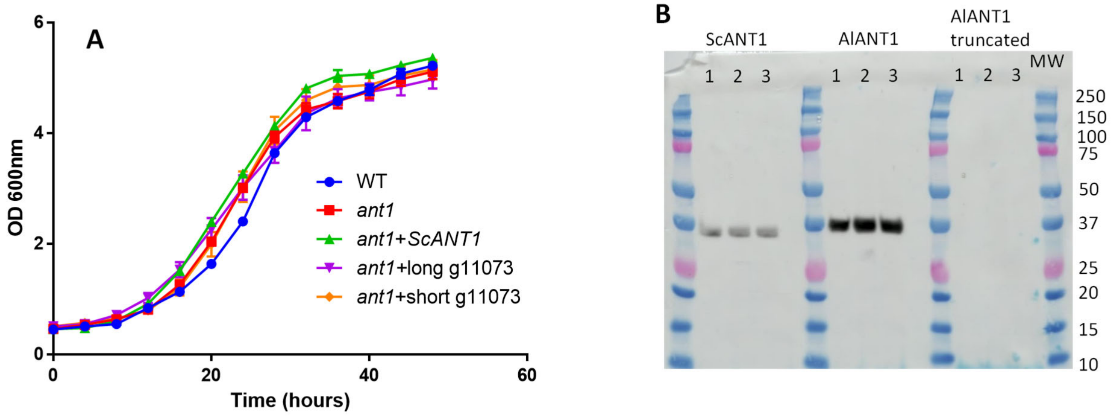

Figure S3: Complemented strains of *Saccharomyces cerevisiae Δant1*. All the plasmids incorporated in the different strains were extracted and sequenced for validation. A) growth curves of WT, *Δant1* and the three *Δant1* complemented strains in the presence of glucose. B) Western blot showing the presence of the complemented proteins in protein extracts from different clones of the three complemented strains (three independent clones per construct). For *Δant1* complemented with the truncated form of g11073, none of the selected clones expressed the truncated g11073 protein.
